# Polymorphism Control of Layered MoTe_2_ through Two-Dimensional Solid-Phase Crystallization

**DOI:** 10.1038/s41598-019-45142-x

**Published:** 2019-06-19

**Authors:** Jyun-Hong Huang, Hao-Hua Hsu, Ding Wang, Wei-Ting Lin, Chun-Cheng Cheng, Yao-Jen Lee, Tuo-Hung Hou

**Affiliations:** 1 Department of Electronics Engineering and Institute of Electronics, National Chiao Tung University, Hsinchu, 300 Taiwan; 2 Advanced Technology Research Center, AU Optronics Corporation, Hsinchu, 300 Taiwan; 3 National Nano Device Laboratories, Hsinchu, 300 Taiwan; 4 Department of Physics, National Chung Hsing University, Taichung, 402 Taiwan

**Keywords:** Synthesis and processing, Two-dimensional materials, Synthesis and processing, Two-dimensional materials

## Abstract

Two-dimensional (2D) molybdenum ditelluride (MoTe_2_) exhibits an intriguing polymorphic nature, showing stable semiconducting 2H and metallic 1T′ phases at room temperature. Polymorphism in MoTe_2_ presents new opportunities in developing phase-change memory, high- performance transistors, and spintronic devices. However, it also poses challenges in synthesizing homogeneous MoTe_2_ with a precisely controlled phase. Recently, a new yet simple method using sputtering and 2D solid-phase crystallization (SPC) is proposed for synthesizing high-quality and large-area MoTe_2_. This study investigates the polymorphism control of MoTe_2_ synthesis using 2D SPC. The Te/Mo ratio and oxygen content in the as-sputtered films correlate strongly with the final phase and electrical properties of SPC MoTe_2_. Furthermore, the SPC thermal budget may be exploited for stabilizing a deterministic phase. The comprehensive experiments presented in this work demonstrate the versatile and precise controllability on the MoTe_2_ phase by using the simple 2D SPC technique.

## Introduction

Two-dimensional (2D) molybdenum ditelluride (MoTe_2_) shows a moderate bandgap close to that of Si^1^, high carrier mobility^2,3^, and a large spin-orbit coupling splitting in the valence band^4^. These unique properties are being explored for various electronics^5–7^, optoelectronics^8^, and spintronics^9,10^ applications. In contrast to most 2D transition metal dichalcogenides (TMDs) with only one monotonous phase stable at room temperature, 2D MoTe_2_ exhibits intriguing polymorphism with a low phase transition barrier^11^, and it can be directly synthesized into either a semiconducting or metallic phase^5,12–14^. In the semiconducting 2H phase, the Mo and Te atoms are arranged in in-plane hexagonal symmetry and out-of-plane trigonal prismatic coordination with a space group of P6*m*2. In the metallic 1T′ phase, they are arranged in in-plane monoclinic symmetry and out-of-plane octahedral coordination with a space group of P12_1_/*m*1^15,16^. Both 2H and 1T′ phases not only are stable at room temperature, but also show robust thermal stability up to 815 and 900 °C, respectively^17^. Chemical vapor transportation (CVT)^2^ is a popular method for synthesizing both 2H- and 1T′-MoTe_2_ bulk materials by recrystallizing mixed Mo and Te powders at high temperatures (1100 °C for tens of hours). The cooling rate of the sample determines the final stable phase to be 2H or 1T′. The transition between 2H and 1T′ phases after synthesis could be facilitated by supplying external energy through photons^6^, electrons^18^ and strains^19^. The ability of phase transition was recently explored for forming a lateral 1T′/2H heterophase junction in field-effect transistors (FETs) where the source/drain regions are selectively transformed into the 1T′ phase to reduce the contact resistance^6,20^. However, synthesizing high-quality 2D MoTe_2_ with a controllable phase directly on large-area substrates remains extremely challenging by using conventional chemical vapor deposition (CVD). This is attributed to the weak bonding energy between Mo and Te atoms^5^, and also the aforementioned low transition barrier between the 1T′ and 2H phases. The process window of CVD, depending on the CVD catalyst^21^, Te partial pressure^12,13,22^, Mo precursors^5^, and turbulent flow control^23^, is limited for MoTe_2_ compared with that for other widely available TMDs such as MoS_2_, MoSe_2_, WS_2_, and WSe_2_. The lack of a reliable large-area synthesis process greatly hinders the practical use of MoTe_2_.

Recently we demonstrated a new yet simple synthesis route for large-area, high-quality layered MoTe_2_ by using a sputtering deposition followed by solid-phase crystallization (SPC)^24^. The Mo and Te elements were directly supplied from a compound sputter target without complex chemical reactions and reactant transferring. Because of the physical bombardment of sputtering, the clustering of Mo and Te elements was random, and SPC was necessary to transform as-sputtered amorphous MoTe_2_ thin films into layered structures. To prevent oxidation and decomposition of MoTe_2_ during SPC at high temperatures, the as-sputtered MoTe_2_ was first encapsulated using a SiO_2_ capping layer before SPC, and SPC can be performed easily in a Te-free atmosphere. More comprehensive discussion on the SPC mechanism and electrical characteristics can be found in ref.^24^. We also found that synthesizing homogeneous 1T′- and 2H-MoTe_2_ is possible by controlling the SPC dwell time^24^. However, the polymorphism control of SPC MoTe_2_ is scientifically interesting and requires more comprehensive investigations.

In this work, the polymorphic SPC of MoTe_2_ is studied depending on the composition of the as-sputtered MoTe_2_ and the SPC thermal budget. The composition of the as-sputtered MoTe_2_ was verified using X-ray photoelectron spectroscopy (XPS), and it was found that the composition can be modulated by varying the sputtering temperatures while using an identical MoTe_2_ sputter target. MoTe_2_ is known to be prone to oxidation^17,25^. The ambient oxygen is easily adsorbed on the defective MoTe_2_ surface and leads to significant changes in optical and electrical properties^25^. We found that the Te/Mo ratio and oxygen content in the as-sputtered film correlated strongly with the phase of SPC MoTe_2_ and the electrical characteristics of MoTe_2_ FETs. Furthermore, SPC temperature and time are critical factors for determining the phase of SPC MoTe_2_. This work provides a complete strategy and experimental database for synthesizing a deterministic MoTe_2_ phase via SPC and paves a way for novel applications such as integrated circuits, phase-change memory^18^, and spintronic devices by exploiting the polymorphic nature of 2D MoTe_2_.

## Results and Discussion

**Figure 1:**
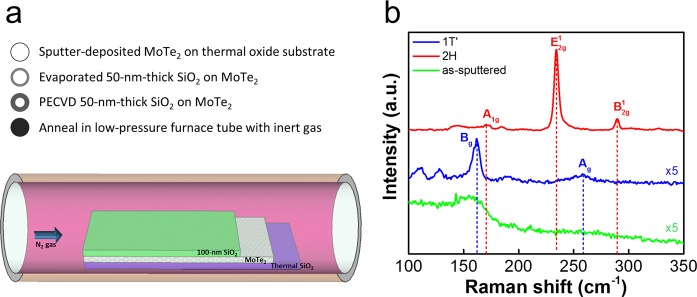
(**a**) SPC synthesis flow of large-area, high-quality MoTe_2_, and (**b**) Raman spectra of as-sputtered MoTe_2_, 2H-MoTe_2_ SPC at 650 °C for 24 h, and 1T′-MoTe_2_ SPC at 850 °C for 1 h.

**Figure 2:**
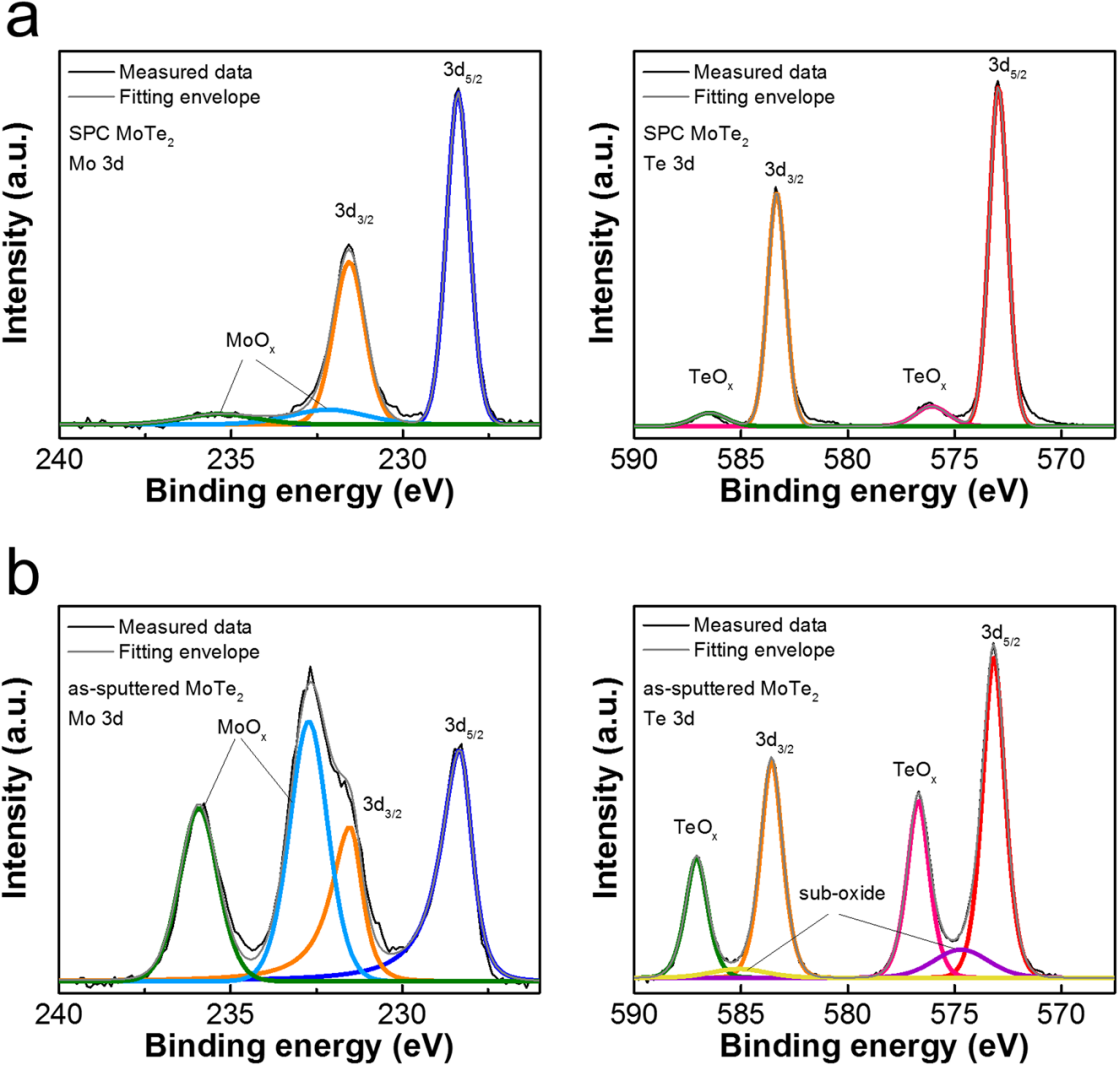
Oxidation effect in MoTe_2_. XPS spectra of Mo 3d and Te 3d core levels in (**a**) SPC crystalline 2H-MoTe_2_ and (**b**) as-sputtered amorphous MoTe_2_. The deconvolution fitting was performed to identify distinct binding energy peaks of Mo (3d_5/2_, 3d_3/2_), Te (3d_5/2_, 3d_3/2_), and their corresponding oxidized states.

The oxidation effects on the as-sputtered and SPC MoTe_2_ were first examined using XPS. The amorphous MoTe_2_ film with a thickness of 5.6 nm was sputtered at 200 °C by using a MoTe_2_ target, and split into two pieces. One was examined by XPS directly (as-sputtered sample). The other (SPC sample) was immediately transferred to an E-gun evaporation chamber to deposit a 50-nm-thick SiO_2_ layer, followed by another 50-nm-thick plasma-enhanced CVD (PECVD) SiO_2_ layer. This composite capping layer prevents oxidation and decomposition of MoTe_2_ in the subsequent SPC process^24^. The MoTe_2_ thin film encapsulated in SiO_2_ was then annealed in a low-pressure furnace in N_2_ to transform into crystalline MoTe_2_ via SPC. The oxide capping was then removed using a dilute HF solution prior to the XPS analysis. More details about the SPC MoTe_2_ formation can be found in the Experimental Section. Figure 1 summarizes the SPC synthesis flow and the typical Raman spectra of as-sputtered MoTe_2_, 2H-MoTe_2_ (SPC at 650 °C for 24 h) and 1T′-MoTe_2_ (SPC at 850 °C for 1 h). The characteristic 2H-MoTe_2_ spectra peak at 171.4 (${A}_{1g}$), 234.7 (${E}_{2g}^{1}$), and 290.0 (${B}_{2g}^{1}$) cm^−1^; those of 1T′-MoTe_2_ spectra peak at 163 (${B}_{g}$), and 262 (${A}_{g}$) cm^−1^. The main peaks of 2H-MoTe_2_ and 1T′-MoTe_2_ are consistent with those reported in the literature^2,5,12,24^. Figure 2 shows the measured binding energy spectra of Mo and Te 3d core levels in the as-sputtered and SPC 2H-MoTe_2_ samples. The core-excitation states of 3d_5/2_ and 3d_3/2_ were separated by 3.2 eV and 10.4 eV for the Mo and Te valence states, respectively. For the SPC sample (Fig. 2(a)), the binding energy spectra of Mo 3d_5/2_ and Te 3d_5/2_ peaked at 228.3 eV (blue curve) and 572.9 eV (red curve), respectively. The calculated Te/Mo ratio was approximately 2.13, in good agreement with a nearly stoichiometric MoTe_2_. For the as-sputtered sample (Fig. 2(b)), the binding energy values of the Mo 3d_5/2_ and Te 3d_5/2_ peaks were in line with those of SPC. However, the asymmetric deconvolution envelopes of Mo 3d_5/2_ (blue curve) and Mo 3d_3/2_ (orange curve) were absent in 2H-MoTe_2_. The similar asymmetric envelops were previously observed in a mixed composite of Mo–Te with oxidized metal^26,27^ or 1T′-MoTe_2_^26,28–30^. The oxidation peaks of Mo 3d_5/2_ and Te 3d_5/2_ were substantial at 232.68 eV (light blue curve) and 576.68 eV (pink curve). By contrast, the Mo–O and Te–O peaks in 2H-MoTe_2_ were significantly reduced, and this is attributed to the recrystallization of Mo–Te bonds at high temperature. The encapsulated structure contained the Mo and Te atoms from out-diffusion and facilitated Mo–Te bonding during SPC^24^. To elucidate the origin of oxidation in as-sputtered MoTe_2_, a much thicker MoTe_2_ (56 nm thick) was sputtered to investigate its depth profiling of composition via XPS, as shown in Fig. S1. The characteristic oxygen 1 s peaks were observed only at the film surface, excluding the possibility of oxygen incorporation during the sputtering deposition. The surface oxidation of the as-sputtered MoTe_2_ was mainly due to the exposure to ambient air at room temperature. We expect a similar but potentially weaker surface oxidation effect would be unavoidable even when the as-sputtered film is immediately encapsulated by the SiO_2_ capping layer because the sample has to be transferred in air between the sputter and evaporator chamber in the present experimental setup.

**Figure 3:**
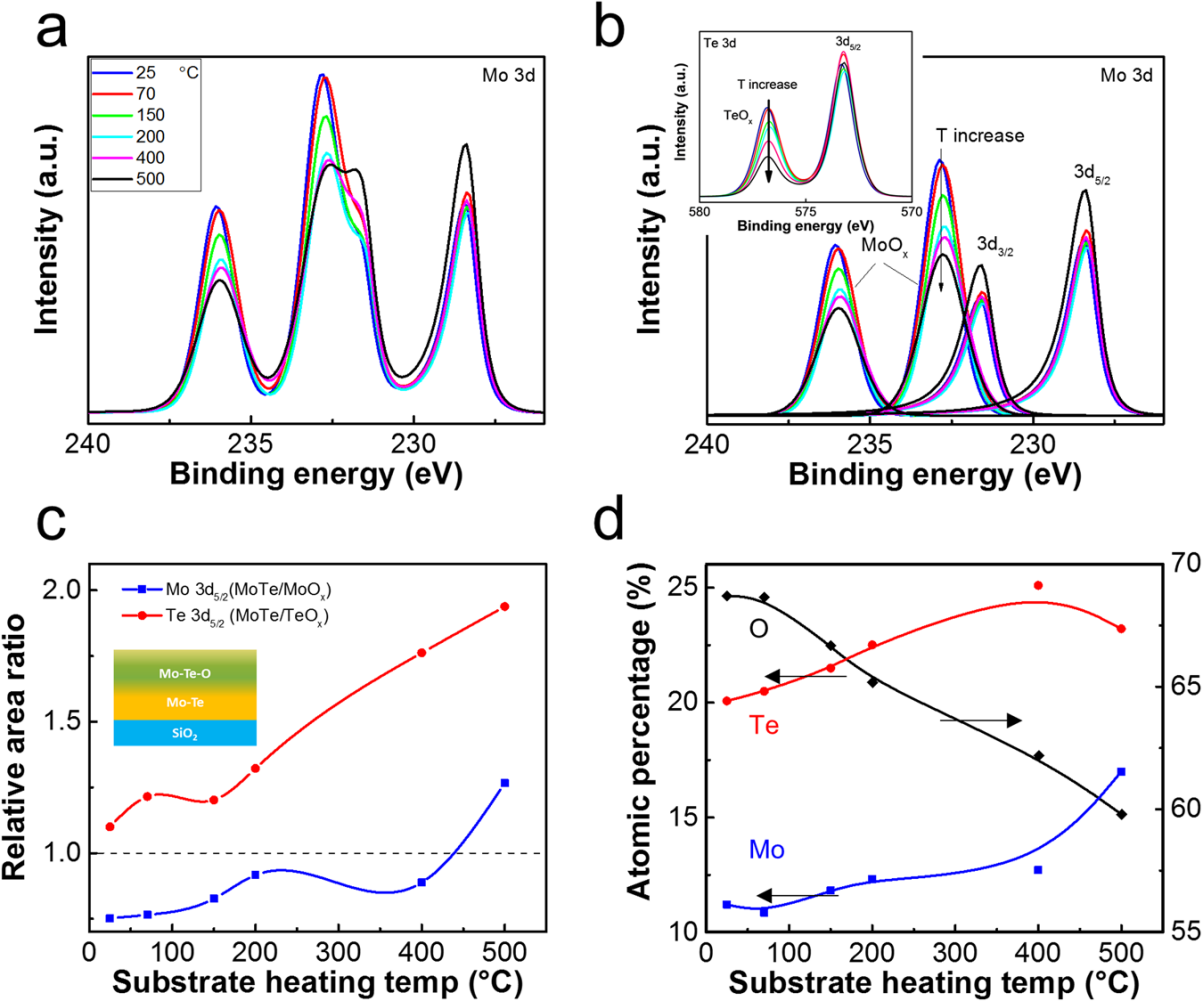
Composition of as-sputtered MoTe_2_. XPS spectra of (**a**) Mo 3d convolution peaks and (**b**) Mo 3d deconvolution peaks from as-sputtered MoTe_2_ films deposited at various temperatures. Inset in (**b**) shows the corresponding XPS spectra of Te 3d. (**c**) The ratios of the integrated area calculated from the Mo 3d_5/2_ (~228 eV), Te 3d_5/2_ (~573 eV), MoO_x_ 3d_5/2_ (232.6 eV), and TeO_x_ 3d_5/2_ (576.6 eV) deconvolution peaks in (**b**). The degree of oxidation depends strongly on the substrate heating temperature. (**d**) Atomic percentages of Mo, Te and O atoms. The amount of oxygen in MoTe_2_ decreased with increasing substrate heating temperature while the ratio of Te/Mo remained nearly constant except for that at 500 °C. The reduction of Te/Mo ratio at 500 °C was due to the Te sublimation.

**Figure 4:**
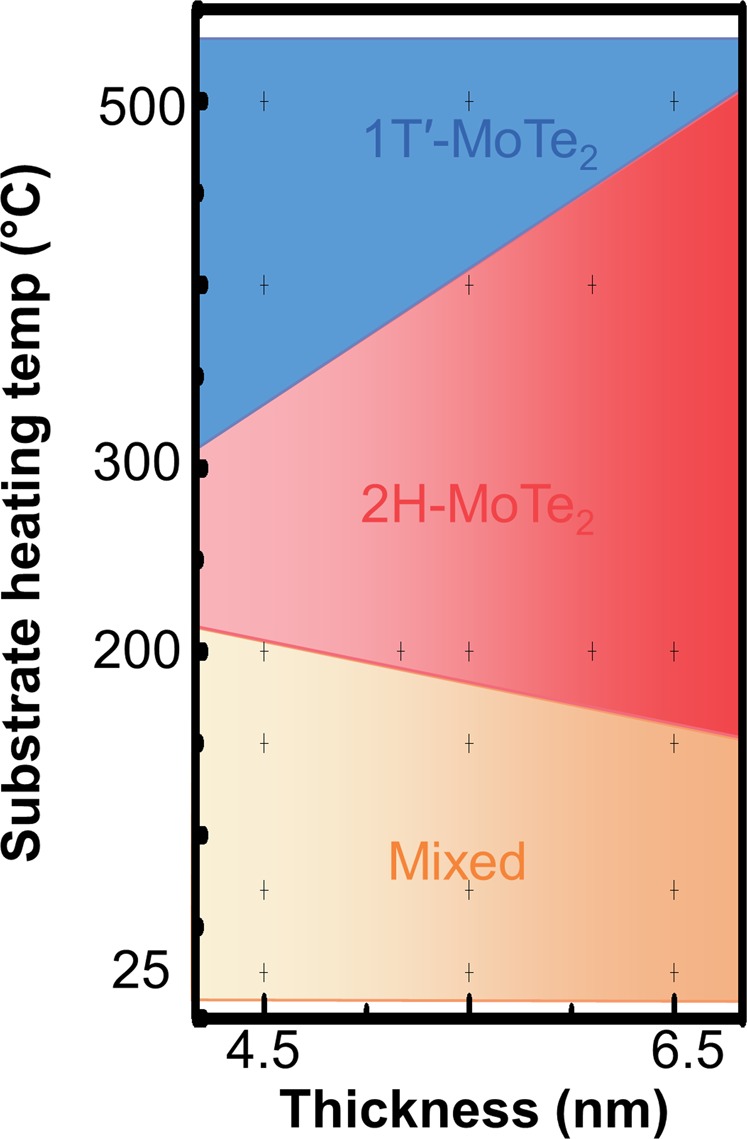
Polymorphic SPC phase diagram as function of substrate heating temperature and thickness of as-sputtered MoTe_2_. The SPC was performed at 650 °C for 24 h for all samples. When sputtered at 500 °C, the 1T′ phase after SPC is attributed to the Te-deficient composition due to Te sublimation. Below 150 °C, mixtures of 1T′ and 2H phases were observed because excessive oxygen might retard the nucleation and recrystallization of 2H-MoTe_2_. Homogeneous 2H-MoTe_2_ after SPC was obtained between 200 to 400 °C depending on the MoTe_2_ thickness.

Substrate heating temperature is known to be an effective knob for engineering the composition, texture, roughness, crystallinity, and nucleation of sputtering films^31,32^ by providing additional migration energy of atoms at the surface. Therefore, we further investigated the SPC effect of MoTe_2_ films sputtered at different substrate temperatures from 25 to 500 °C. Figure S2 shows their respective fittings of Raman spectra. Similar to that shown in Fig. 1, the as-sputtered MoTe_2_ remained mostly amorphous with no strong characteristic peaks corresponding to crystalline MoTe_2_. The weak 1T′ characteristic peaks and the asymmetric Mo 3d envelopes shown in Fig. 2(b) suggest possible formation of some 1T′ nucleation sites in as-sputtered films. However, the thermal energy provided during sputtering was insufficient to facilitate the recrystallization of MoTe_2_. The Raman intensity degraded when sputtered at 500 °C and above, indicating a decomposition of films. On the other hand, XPS spectra of Mo 3d core levels in Fig. 3(a) show that the substrate temperature correlated strongly with the film composition even when the identical sputter target was used. Figure 3(b) shows the deconvolution of XPS spectra designated to the Mo–Te and Mo–O bonds. The intensity of the Mo–O peak decreased with increasing substrate temperature while the change on the Mo–Te peak was relatively small except for the sample sputtered at 500 °C. The inset of Fig. 3(b) shows a similar phenomenon for the XPS spectra of Te 3d core levels. Figure 3(c) shows the ratio of the integrated area between composite states (Mo–Te) and oxidation states (Mo–O, Te–O). The relative areas of Mo–Te gradually increased with temperature. The MoTe_2_ films sputtered at high temperatures became more resistant to surface oxidation when exposed to air (inset of Fig. 3(c)). While the exact root cause is still under investigation, two plausible mechanisms are: the bonding strength between Mo and Te is higher or the density of MoTe_2_ is higher when sputtered at high temperatures so that the film is more robust against oxidation or oxygen diffusion. Figure 3(d) shows the calculated atomic percentages of O, Mo, and Te atoms. The amount of oxygen decreased from 70 at.% at room temperature to 59 at.% at 500 °C. The ratio of Te/Mo remained at around 1.8 to 2.0 when sputtered at up to 400 °C. The ratio corresponds to the composition of the sputter target. When sputtered at 500 °C, the Te/Mo ratio decreased to 1.4 because of the low sublimation temperature of tellurium^33,34^. The degree of oxidation also reduced with increasing the film thickness (Fig. S3), i.e. the relative percentage of the oxidized region is less in a thicker film, which supports the surface oxidation effect. The phase of MoTe_2_ after SPC depends strongly on the composition of the as-sputtered film. SPC at 650 °C for 24 h was performed on MoTe_2_ sputtered at different temperatures and with different thicknesses ranging from 4.5 to 6.5 nm. The final phase diagram confirmed by Raman analysis is summarized in Fig. 4. It is worth noting that different from the previous phase diagram reported for the MoTe_2_ bulk crystals synthesized using CVT^2^, the results here consider the effects of 2D SPC in ultrathin films and additional oxygen incorporation. Three distinct regions of 1T′, 2H, and mixed 1T′-2H phases were observed. For MoTe_2_ sputtered at high temperatures around 500 °C, Te-deficient composition due to Te sublimation stabilized the 1T′ phase after SPC^2^. For those sputtered at temperatures below 500 °C, the nearly stoichiometric MoTe_2_ composition favored the 2H phase^2^. However, for those sputtered at temperatures below 150 °C, homogenous 2H-MoTe_2_ was difficult to obtain across a large area. A mixture of 1T′ and 2H phases was observed. A three-step process for recrystallizing 2H-MoTe_2_ was previously reported^24^. Amorphous as-sputtered MoTe_2_ is first crystallized homogeneously into 1T′-MoTe_2_. The preferential 1T′-MoTe_2_ formation is possibly due to the favorable strain condition in the encapsulated structure^19^, abundant local tellurium vacancies in the amorphous film^12^, or potential formation of 1T′ nucleation sites aforementioned. As the SPC progresses, a few nucleation sites of 2H-MoTe_2_ start to develop in localized Te-rich regions. Finally, the entire 1T′-MoTe film is recrystallized into a homogeneous 2H-MoTe_2_ phase from the nucleation sites. Therefore, the mixed 1T′-2H phase could be attributed to the retardation of the 2H-MoTe_2_ nucleation or recrystallization at the presence of excessive surface oxidation when sputtering MoTe_2_ at low temperatures. Thinner MoTe_2_ is more prone to both Te sublimation and surface oxidation, and thus the stable 2H-MoTe_2_ region in Fig. 4 shrunk substantially as the thickness of MoTe_2_ reduced.

**Figure 5:**
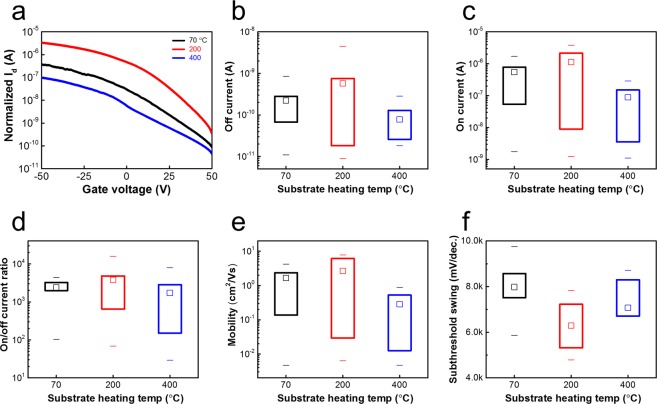
2H-MoTe_2_ back-gated transistor. The SPC layered 2H-MoTe_2_ were sputtered at 70, 200 and 400 °C, respectively. (**a**) Typical current-voltage transfer characteristics. The current was normalized to unit channel length and width. (**b**–**f**) Statistical off current, on current, on/off current ratio, mobility, and subthreshold swing of devices, respectively. At least 14 random devices were measured. The channel length (width) ranges from 4(32) μm to 30(100) μm. The measured electrical data were normalized to the unit length and width for fair comparison. The non-monotonic dependence on the sputtering temperature is related to the p-doping effect of oxygen incorporation.

To further correlate the relations among composition, SPC growth, and final electrical properties, back-gated transistors were fabricated on the 2H-MoTe_2_ samples sputtered at different temperatures. All samples received the same SPC annealing at 650 °C for 24 h. 25-nm Pd metal was used as the source/drain contacts. Figure 5(a) illustrated typical current-voltage transfer characteristics. All the devices exhibited p-type conduction characteristics, but that sputtered at 200 °C presented the strongest p-type conduction and best electrical performance. In Fig. 5(b–f), statistical plots of the major device parameters showed a consistent trend. The field-effect hole mobility μ_h_ in the linear region was extracted using μ_h_ = (d*I*_D_/d*V*_G_)(*L*/*W*)(1/*V*_D_*C*_G_) at *V*_D_ = −1 V where *L*, *W*, and *C*_G_ represent the channel length, channel width, and gate capacitance per unit area, respectively. The extracted mobility from the device sputtered at 200 °C was comparable with those reported for CVT MoTe_2_^2,6,8^. The non-monotonic dependence of device performance on the sputtering temperature might be related to the oxygen doping effect. Oxygen incorporation is known to induce p-type doping in MoTe_2_^35^. A suitable amount of oxygen introduced by surface oxidation presented a negligible adverse effect of SPC retardation while providing necessary p-type doping. By contrast, the intrinsic MoTe_2_ obtained from the CVT method with minimal defects usually exhibits ambipolar conduction^7,35,36^. Precisely controlling oxygen content in MoTe_2_ is critical for enabling p-type conduction even when both the MoTe_2_ and SiO_2_ capping are deposited in the same chamber with no intentional air exposure in between.

**Figure 6:**
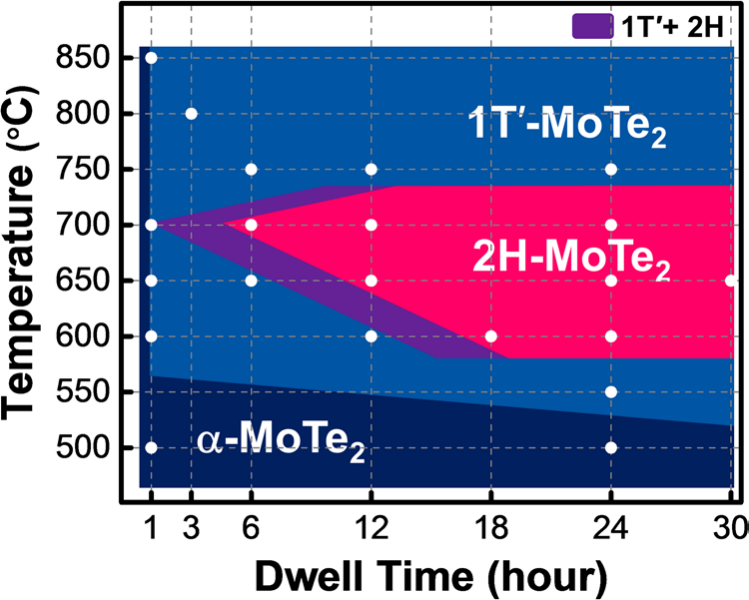
Polymorphic SPC phase diagram as function of SPC temperature and dwell time. The identical 6.5-nm-thick MoTe_2_ sputtered at 200 °C was used. 1T′ phase was predominant at 850 °C and 500–600 °C. For SPC temperatures between 600–700 °C, the evolution from the 1T′ phase to 2H phase was progressed with dwell time. For SPC temperatures at 500 °C and below, the film remained amorphous.

Figure 6 shows the effect of SPC temperature and dwell time on the polymorphic phase diagram of MoTe_2_. The same MoTe_2_ sample with a thickness of 6.5 nm sputtered at 200 °C was split into smaller pieces, and then these pieces were subjected to SPC at 500 °C to 850 °C for 1 h to 24 h. Three distinct regions, the 2H phase (red background), 1T′ phase (blue background), and amorphous phase (dark blue background), were observed. For a high SPC temperatures at 850 °C, 1T′ phase was preferable^2^. For SPC temperatures between 600–700 °C, the aforementioned evolution from the 1T′ to 2H phase can be observed with increasing the dwell time. For SPC temperatures below 600 °C but above 500 °C, the thermal energy was insufficient for the nucleation of 2H-MoTe_2_, and the MoTe_2_ stayed at its 1T′ phase. For SPC temperatures at 500 °C and below, the thermal energy was even less, and the recrystallization into either 1T′- or 2H-MoTe_2_ was prohibited. Therefore, the MoTe_2_ remained at the amorphous phase regardless of the dwell time.

## Conclusions

We studied the polymorphic control of MoTe_2_ synthesis using 2D SPC. The surface oxidation of as-sputtered MoTe_2_ depends strongly on the substrate heating temperature during sputtering. The tradeoff of surface oxidation is present. Excessive oxidation retards the formation of homogeneous 2H-MoTe_2_, but an appropriate amount of oxygen doping enhances the p-type doping and thus the device performance. Furthermore, SPC thermal budgets depending on the temperature and time may be exploited for stabilizing a deterministic phase of large-area layered MoTe_2_. The 2D SPC technique demonstrates versatile controllability on the MoTe_2_ phase and presents new opportunities on researching MoTe_2_-based devices.

## Experimental Section

### MoTe_2_ sputtering and SPC

Ultra-high vacuum DC magnetron sputtering system with a base pressure of 10^−8^ torr was utilized for depositing MoTe_2_ films. The films were sputtered on a SiO_2_ substrate (300-nm-thick SiO_2_ on Si) using a high-purity MoTe_2_ target (99.9% purity; Kojundo Chemical Laboratory), an Ar flow of 20 sccm, a working pressure of 10 mTorr, and DC power of 50 watts. The substrate was heated to various temperatures between 25 to 500 °C using a chuck heater and a ramp rate of 31 °C per minute. For the SPC of MoTe_2_, the as-sputtered MoTe_2_ sample was unloaded from the sputter chamber and transferred in air immediately to another separate electron-gun evaporator to deposit a 50-nm-thick SiO_2_ capping layer by using a source of SiO_2_ granules, followed by another 50-nm-thick SiO_2_ layer deposited by PECVD using SiH_4_ and N_2_O as precursors at 300 °C. The composite SiO_2_ layer suppresses oxidation and decomposition of MoTe_2_ in the subsequent annealing. The evaporated SiO_2_ layer prevents potential plasma damage or oxidation on the as-deposited films during PECVD while the PECVD SiO_2_ is dense and of favorable quality^24^. The encapsulated MoTe_2_ sample was then annealed for SPC in a low-pressure furnace at 30 Torr and 650 °C for 24 h in N_2_. The SiO_2_ capping layer was removed using dilute HF (1.2%) before the XPS analysis of MoTe_2_ after SPC.

### Properties characterization

Raman spectra were collected in a spectrograph system (Andor Technology; model: SR-500i-D2-R) with a high-performance laser source (Cobolt Samba^TM^; wavelength 532 nm and laser power of 5 mW) and CCD detector (model: DV416A-LDC-DD). The silicon vibration peak located at 520 cm^−1^ was utilized to calibrate the measurement system. The elemental composition was determined using XPS (Thermo Fisher Scientific Theta Probe, Al Kα X-ray source). The carbon 1 s peak was used to calibrate the reference of binding energy. The measurement was performed in an ultra-high vacuum condition (~10^−9^ torr) and with a beam size of 400 μm. For XPS depth profiling, *in-situ* Ar sputtering was utilized with an etching rate of 0.298 nm per second for the as-sputtered MoTe_2_ film. The spectra of binding energy were deconvoluted using the Shirley background correction and characterized based on *NIST XPS database*^24^. The relative sensitivity factors of Mo 3d_5/2_, Te 3d_5/2_, and O 1 s were 6.51, 25 and 2.88, respectively. The deconvoluted Mo 3d line shape was fit using an asymmetry distribution of LA (1.1, 2.3, 2) while others were fit using a symmetric distribution of Gaussian–Lorentzian (60).

### Fabrication of back-gated transistors

To fabricate back-gated transistors, negative photoresist was spin-coated on the SPC MoTe_2_ sample and then baked on a hot plate at 90 °C for 60 s. The contact regions were defined through a mask in a contact aligner using the hard-contact mode and an exposure time of 3 s (light source: NUV, 300 nm–400 nm, 1000 W). After the photoresist development, the sample was baked at 100 °C for 120 s on a hot plate. The SiO_2_ capping layer on the contact regions was removed using a dilute HF solution (1.2%). Then, 25-nm-thick Pd metal was deposited using an E-gun evaporator and lifted off in acetone followed by DI water rinse. Finally, the active channel region was defined using the similar photolithography procedure for the contact region and plasma dry etched in Ar + CHF_3_ + SF_6_. The device characteristics of back-gated FETs were measured at room temperature by using a HP-4156B semiconductor parameter analyzer.

## Supplementary information

SUPPLEMENTARY INFORMATION
