# Supplementary material for: Polymorphism Control of Layered MoTe2 through Two-Dimensional Solid-Phase Crystallization
Source: Sci Rep. 2019 Jun 19;9:8810. doi: 10.1038/s41598-019-45142-x (PMC6584554; doi:10.1038/s41598-019-45142-x)
Supplement: Supplementary file 1 — SUPPLEMENTARY INFORMATION [file 41598_2019_45142_MOESM1_ESM.pdf]

Supporting Information

Polymorphism Control of Layered $MoTe_2$ through Two-Dimensional Solid-Phase Crystallization

Jyun-Hong Huang[1], Hao-Hua Hsu[1], Ding Wang[2], Wei-Ting Lin[2], Chun-Cheng Cheng[2], Yao-Jen Lee[2,3], and Tuo-Hung Hou[1]

[1]Department of Electronics Engineering and Institute of Electronics, National Chiao Tung University, Hsinchu 300, Taiwan

[2]Advanced Technology Research Center, AU Optronics Corporation, Hsinchu 300, Taiwan

[3]National Nano Device Laboratories, Hsinchu 300, Taiwan

[4]Department of Physics, National Chung Hsing University, Taichung 402, Taiwan

Correspondence and requests for materials should be addressed to T-H Hou

E-mail: thhou@mail.nctu.edu.tw

S1. Depth profile analysis of as-sputtered $MoTe_2$

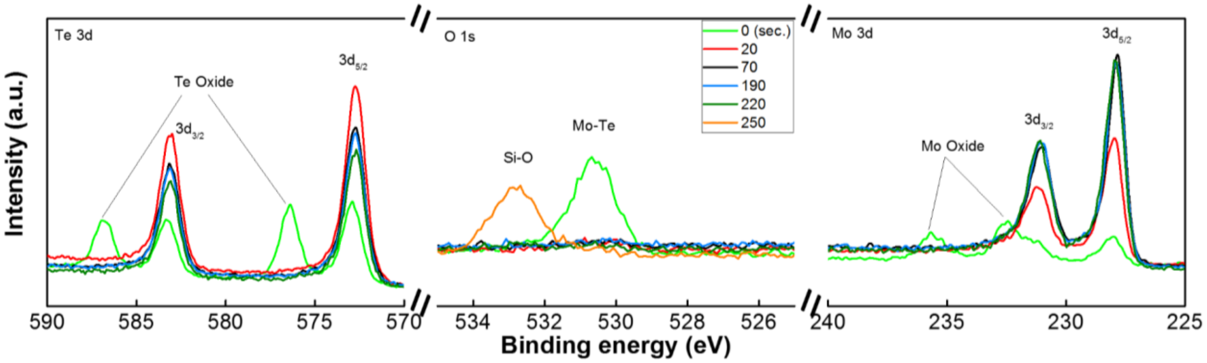

Figure S1. XPS depth analysis of 56-nm-thick $MoTe_2$.

In order to verify when and where oxygen incorporation occurred in the as-deposited $MoTe_2$, we performed XPS depth profiling for a 56-nm-thick $MoTe_2$. The $MoTe_2$ film was deposited at 25 ˚C. The film thickness was identified using a surface profiler. The calculated etching rate was 0.298 nm per second by using in-situ Ar sputtering in the XPS chamber. Only surface oxidation was observed, suggesting that the as-deposited $MoTe_2$ was oxidized in air rather than inside the sputtering chamber.

S2. Raman spectra of $MoTe_2$ sputtered at various substrate temperatures

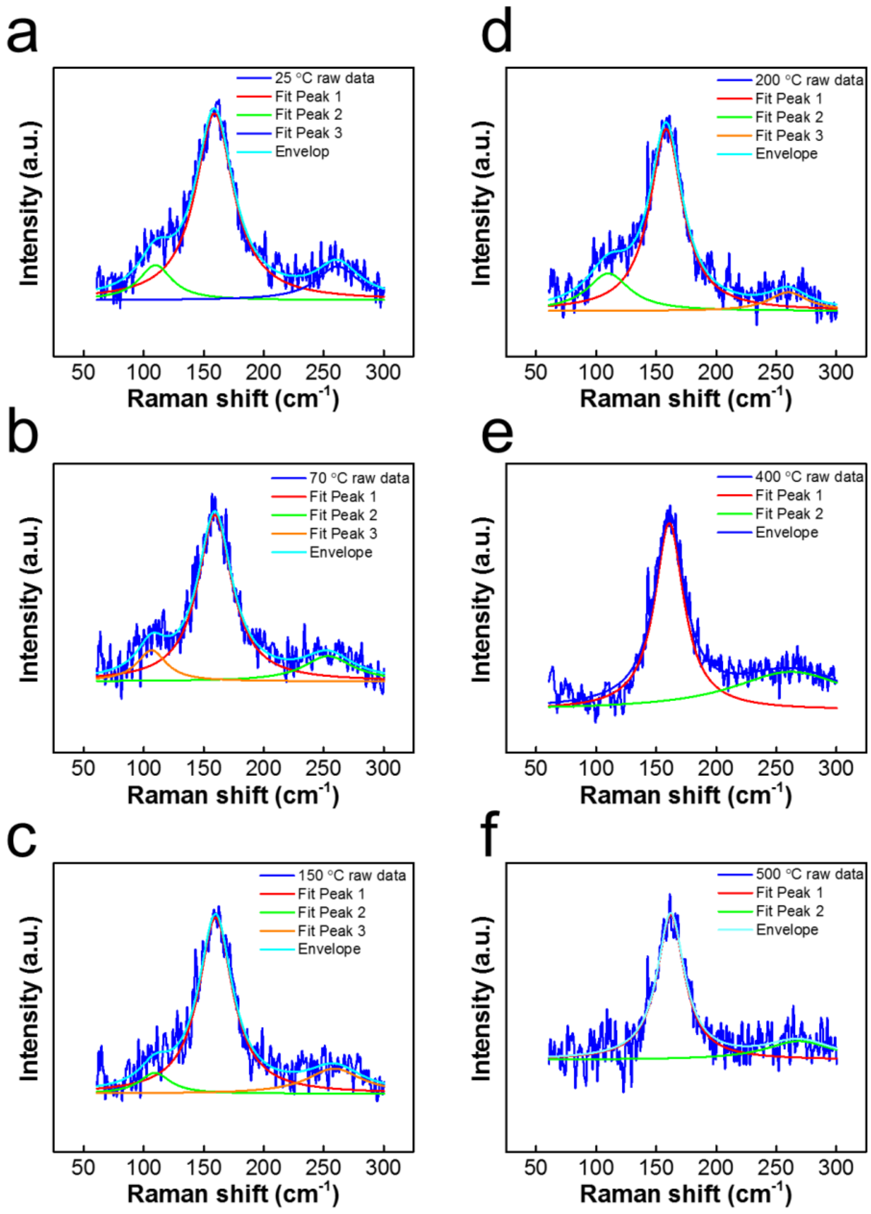

Figure S2. Raman spectra for $MoTe_2$ sputtered at 25 ˚C, 70 ˚C, 150 ˚C, 200 ˚C, 400 ˚C, and 500 ˚C, respectively. (a-f) Deconvoluted Raman peaks showing 1 T′-like signals but with extremely weak intensity.

The Raman measurement did not reveal any apparent relation for $MoTe_2$ sputtered at different temperatures ranging from 25 to 500 ˚C. From the curve fitting, weak 1T′ characteristic peaks are

likely present, which is consistent with the result of the asymmetric envelope of Mo 3d in the XPS analysis. On the other hand, in-situ recrystallization cannot be easily accomplished because Te easily sublimes at temperatures above 450 ˚C[1,2]. As a result, the Raman intensity degraded for films sputtered at 500 ˚C.

S3. Thickness-dependent surface oxidation of as-deposited $MoTe_2$

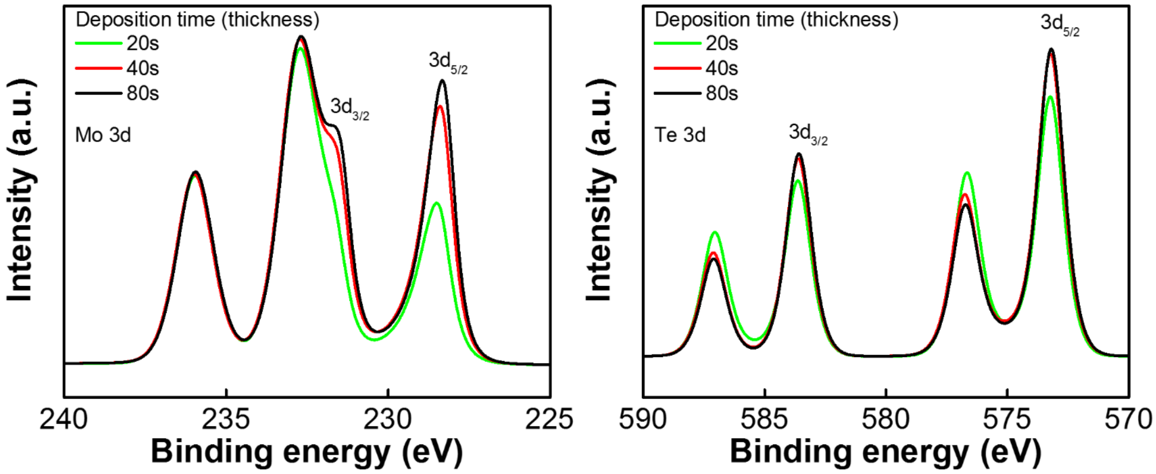

Figure S3. Surface oxidation of as-sputtered $MoTe_2$ with various deposition times of 20 s, 40 s, and 80 s, respectively, at 200 ˚C. The peak intensity of Te–O decreased with the film thickness.

According to the XPS depth profiling analysis in S1, a thicker $MoTe_2$ film was expected to possess stronger Mo–Te signals because the oxidation occurred only at the surface. The $MoTe_2$ samples were sputtered at 200 ˚C for 20 s, 40s, and 80 s, respectively, and then analyzed using XPS. The reduced Te–O peak intensity with the film thickness was consistent with the assumption.

Reference

1 Ohsugi, I. J., Tokunaga, D., Kato, M., Yoneda, S. & Isoda, Y. Dissociation and sublimation of tellurium from the thermoelectric tellurides. *Mater. Res. Innov.* **19**, S5-301-S305-303, doi:10.1179/1432891714z.0000000001097 (2015).

2 Riley, B. J., Johnson, B. R., Schaef, H. T. & Sundaram, S. K. Sublimation–condensation of multiscale tellurium structures. *J. Phys. Chem. C* **117**, 10128-10134, doi:10.1021/jp400363a (2013).
